# Flow cytometric analysis of platelet surface glycoproteins in the diagnosis of thirty-two Turkish patients with Glanzmann thrombasthenia: a multicenter experience

**DOI:** 10.3906/sag-2006-107

**Published:** 2021-08-30

**Authors:** Berkay SARAYMEN, Sabahattin MUHTAROĞLU, Mustafa Yavuz KÖKER, Nazan SARPER, Emine ZENGİN, Canan ALBAYRAK, Davut ALBAYRAK, Bülent ZÜLFİKAR, Başak KOÇ ŞENOL, Esma BENTLİ, Semih YILMAZ, Aysun ÇETİN, Bülent ESER, Mustafa ÇETİN

**Affiliations:** 1 ERNAM-Nanotechnology Research and Application Center, Erciyes University Kayseri Turkey; 2 Department of Biochemistry, Faculty of Medicine, Erciyes University, Kayseri Turkey; 3 Department of Immunology, Faculty of Medicine, Erciyes University, Kayseri Turkey; 4 Department of Pediatric Hematology, Faculty of Medicine, Kocaeli University, Kocaeli Turkey; 5 Department of Pediatric Hematology-Oncology, Faculty of Medicine, Ondokuz Mayis University, Samsun Turkey; 6 Department of Pediatric Hematology-Oncology, Samsun Medical Park Hospital, Samsun Turkey; 7 Department of Pediatric Hematology-Oncology, Oncology Institute, Istanbul University, İstanbul Turkey; 8 Betül-Ziya Eren Genome and Stem Cell Center, Erciyes University, Kayseri Turkey; 9 Department of Agricultural Biotechnology, Seyrani Faculty of Agriculture, Erciyes University, Kayseri Turkey; 10 Department of Hematology, Antalya Medical Park Hospital, Antalya Turkey; 11 Department of Hematology, Faculty of Medicine, Erciyes University, Kayseri Turkey

**Keywords:** Glanzmann thrombasthenia, glycoprotein IIb/IIIa, flow cytometry, platelet-rich plasma

## Abstract

**Background/aim:**

Glanzmann thrombasthenia (GT) is a rare autosomal recessively inherited bleeding disorder characterized by the quantitative (type 1 and type 2) or qualitative (type 3) deficiency in platelet membrane glycoprotein (GP) IIb/IIIa (CD41a/CD61) fibrinogen receptors. In type 1, 2, and 3, CD41a/CD61 expression is 5%, 5%–20% and above 20%, respectively. In this study, diagnosis of GT was confirmed and subgroups were identified in 32 Turkish patients by flow cytometry analysis.

**Materials and methods:**

CD41a/CD61 expression levels in platelet-rich plasma (PRP) obtained from peripheral venous EDTA blood samples were analyzed with a BD FACSCanto II flow cytometer (Becton Dickinson, Franklin Lakes, NJ, USA). GT subgroup analysis was performed by counting 50,000 events in the BD FACSDiva Software v6.1.3 program of the instrument.

**Results:**

In the present study, in blood samples of 32 patients from 23 families with GT and 22 healthy controls, co-expression levels of CD41a and CD61 in PRP was analyzed. 12 out of 23 families were consistent with type 1 GT (52.2%), 4 were consistent with type 2 GT (17.4%), and 7 were consistent with type 3 GT (30.4%).

**Conclusion:**

Especially due to consanguineous marriages, GT with various glycoprotein levels may be detected. As a result of the flow cytometry analysis of the present study with the highest GT patient population in Turkey, type 1 GT patients were the most common subgroup. In the determination of the GT subgroups; especially in the detection of type 3 GT, flow cytometry is the most sensitive glycoprotein analysis method. In addition to light transmission aggregometry, CD41a/CD61 study by flow cytometer confirms diagnosis when mutation analysis cannot be performed.

## 1. Introduction

Glanzmann thrombasthenia (GT) was first described by Eduard Glanzmann in 1918 as “hereditary hemorrhagic thrombasthenia” [1]. GT is an autosomal recessive bleeding disorder. The disorder is characterized in that platelets fail to bind to fibrinogens and are unable to form aggregates in the presence of a range of physiological agonists such as adenosine diphosphate (ADP), thrombin, epinephrine, or collagen. The apparent defect in patients is caused by mutations in the genes (ITGA2B or ITGB3) encoding GP IIb (αIIb) or GP IIIa (β3) proteins of the GP IIb/IIIa complexes on the platelet surface [2–4].

GP IIb/IIIa receptors account for approximately 15% of total proteins on the platelet surface. Although GT is a rare disorder, it is the best known of hereditary disorders of platelet function [4]. The thrombasthenic phenotype is associated with quantitative or qualitative abnormalities of the αIIbβ3 integrin platelet fibrinogen receptor through the incorporation of platelets in the formation of thrombus or aggregate in the area of vascular injury [5]. Thrombasthenic platelets adhere to the affected subendothelial tissue and secrete from depot granules. However, successive reactions of platelets spreading on the affected surface and thrombus formation are damaged [6].

The classification of GT is based on the content of platelet alpha granules, the degree of fibrinogen and clot retraction, and the expression level of CD41a/CD61. GT has three subgroups, including type 1, type 2, and type 3 [5,7,8].

Type 1 GT is the most common subgroup with CD41a/CD61 expression levels below 5% and low levels of fibrinogen in alpha granules of activated platelets that bind poorly to fibrinogen [7,9].

Patients with type 2 GT have a CD41a/CD61 expression level of 5%–20% in their platelets. These patients have significant platelet alpha granule fibrinogen and normal or moderate clot retraction [7]. The presence of the fibrinogen storage pool is consistent with a preserved fibrinogen receptor function. Classification of type 1 and type 2 GT has been an appropriate method for identifying GT patients and comparing clinical symptoms with molecular abnormalities [10].

Type 3 GT (variant type) is defined as patients presenting the clinical phenotype of GT but expressing CD41a/CD61 in amounts greater than 20% that normally support platelet aggregation [7,10,11]. It is characterized by a lack of activated platelets due to antibodies or soluble fibrinogen, which identify factors related to activation on αIIbβ3 integrin. It is often caused by a single amino acid change and is defined by the residual functional response of platelets [10].

GT may manifest itself shortly after birth. It starts with purpura in the newborn, followed by attacks of mucocutaneous bleeding and spontaneous bruising. Most patients are diagnosed before the age of 5 years. Recurrent episodes of epistaxis occur and one of the most common symptoms has been reported to be gingival bleeding. Fatal bleeding episodes occur during a lifetime of a patient with GT, while the prevalence of severe bleeding decreases with age [12]. There is insufficient evidence to support the relationship between gene defect in GT and the severity of bleeding, as affected individuals show different bleeding tendencies, even in the same family or ethnic group [7].

Although the exact number is unknown, one out of every one million individuals is estimated to be GT [13,14]. Studies have shown that GT is more common in women than men probably due to severe menorrhagia female cases are diagnosed. Approximately 58% of GT cases are women and 42% are men [12]. Although GT can be seen in all communities around the world, it is more common in ethnic groups such as Iraqi Jews, Palestinians, and French Gypsies, where consanguinity rates are high [15,16]. 

The aim of our study was to confirm the diagnosis of GT and to determine the subgroups of 32 Turkish GT patients with flow cytometry.

## 2. Materials and methods

### 2.1. Patients and controls

Ambient EDTA blood samples of the patients with GT and control samples were shipped from four pediatric hematology centers (Kocaeli, Samsun, İstanbul, Kayseri) to the laboratory. All patients had mucosal bleeding (epistaxis, gingival bleeding, and/or menorrhagea) requiring treatment. Platelet counts and platelet morphology were normal, bleeding time was prolonged, activated partial thromboplastin time (PTT), prothrombin time, and assessments for von Willebrand disease such as von Willebrand factor (vWF) antigen, ristocetin cofactor activity, and factor VIII coagulant activity were normal. Light transmission aggregometry showed absent aggregation to all agonists other than to ristocetin (ADP, collagen, thrombin, and arachidonic acid). Healthy volunteers who have no coagulapathy and did not use any drug were choosen as controls. During the flow cytometry evaluation, samples of GT patients and control samples were studied in the same run. Mutations in genes (ITGA2B or ITGB3) encoding GP IIb or GP IIIa proteins of GP IIb/IIIa complexes on the platelet surface were detected in all patients. Among those detected, there are c. 1752 + 2T> C (especially in type 1 GT patients), c. 1772A> C (especially in type 2 GT patients), c. 1697G> A (especially in type 3 GT patients), and 5 novel mutations (especially in type 1 GT patients) previously unidentified (data not shown).

### 2.2. Laboratory work-up

Three-mililiter EDTA blood samples were centrifuged at 200 g for 10 min at room temperature and the upper two thirds of the PRP was removed into a seperate tube. PRP was then centrifuged once again at 200 g for 10 min in order to concentrate the platelets further. 

### 2.3. Antibodies and reagents

The monoclonal antibodies including FITC Mouse IgG1κ (clone: MOPC-21), PE Mouse IgG1κ (clone: MOPC-21), PerCP Mouse IgG1κ (clone: MOPC-31C), FITC labeled anti-human CD41a (clone: HIP8), PE labeled antihuman CD42b (clone: HIP1), PerCP labeled antihuman CD61 (clone: RUU-PL7F12), and CellWASH (catalog no:349524, used for cell washing process) were obtained from BD Biosciences (San Jose, CA, USA).

### 2.4. Flow cytometry

Two tubes were prepared for each control and patient sample. 100 µL PRP were incubated with 5 µL of monoclonal antibodies FITC Mouse IgG1κ, PE Mouse IgG1κ, PerCP Mouse IgG1κ in the first tube and 5 µL of monoclonal antibodies FITC labeled anti-human CD41a, PE labeled antihuman CD42b, PerCP labeled antihuman CD61 in the second tube for 15 min at room temperature and in the dark. After incubation, 2 mL of CellWASH was added to the tubes and centrifuged at 3000 g for 5 min at room temperature. Supernatant was discarded carefully and then 200 µL of CellWASH were added to each tube. After that tubes were directly placed on the flow cytometer (FACSCanto II, Becton Dickinson, Franklin Lakes, NJ, USA). Analyzes and interpretations were carried out by counting 50,000 events in the BD FACS Diva Software v:6.1.3 program of the instrument. Fluorescence measurement was evaluated as the percentage of positive cells above a threshold set with the isotype control tube acting as negative control. Compensation for fluorochrome spectral overlap was adjusted for analysis. Control and patient samples were studied in the same run.

### 2.5. Statistical analysis

The datas were obtained using Microsoft Office Excel 2016 program and were expressed as median and minimum-maximum values.

## 3. Results

### 3.1. Clinical and demographic characteristics of patients

Thirty-two patients from 23 families (18 males, 14 females) were included in the study. Patients’ age during the study was 5–28 years (median 12 years). Healthy volunteers (12 males, 10 females, 6–43 years, median 13.5 years) who have no coagulapathy and did not use any drug were controls. Bleeding manifestations included epistaxis in 23 patients, gum bleeding in 18 patients, easy brusing in 22 patients, menorrhagia in 12 patients, bleeding at the injection site in 18 patients, and haematuria in 4 patients. History of consanguinity could be revealed in 20 of 23 families (86.9%). The age and sex distribution of the patient group was similar to the control group. 

### 3.2. Results of flow cytometry analysis

Flow-cytometry analysis of 32 Turkish GT patients from 23 families and 22 control samples were determined at the Erciyes University Mehmet-Kemal Dedeman Hematology-Oncology Hospital Flow Cytometry and Cell Processing Laboratory. 

**Figure 1:**
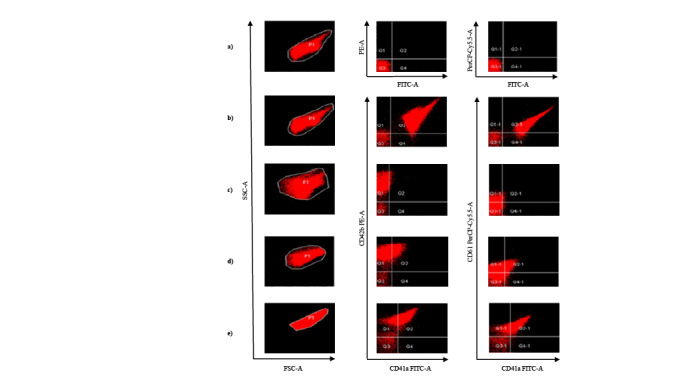
a) Isotype controls with null levels of IgG1κ FITC, IgG1κ PE, and IgG1κ PerCP. b) Control with normal levels of both CD41a FITC, CD42 PE, and CD61 PerCP. c) Type 1 GT with null or reduced levels of both CD41a FITC and CD61 PerCP (<%) and normal levels of CD42 PE. d) Type 2 GT with reduced levels of both CD41a FITC and CD61 PerCP (5%–20%) and normal levels of CD42 PE. e) Type 3 GT with near normal levels of CD41 FITC and CD61 PerCP and normal levels of CD42 PE.

FITC Mouse IgG1κ, PE Mouse IgG1κ, and PerCP Mouse IgG1κ isotype controls were used to analyze each patient and control sample (Figure 1a). CD41a, CD42b, CD61, and CD41a/CD61 expression levels in controls and GT patients were assessed in PRP (Figures 1b–1e).

Expression levels of CD41a, CD42b, CD61 and CD41a/CD61 in control samples were 95.8% to 100% (median: 99.7), 94.3% to 100% (median: 98.9), 93.8% to 100% (median: 99.5), and 93.8% to 100% (median: 99.1), respectively.

In this analysis, 19 out of 32 patients were consistent with type 1 GT (59.4%), 4 were consistent with type 2 GT (12.5%), and 9 were consistent with type 3 GT (28.1%). 

Expression levels of CD41a, CD42b, CD61 and CD41a/CD61 in type 1 GT patients were 0% to 5.2% (median: 0.6), 88.4% to 98.5% (median: 92), 8.6% to 74.7% (median: 21.4), and 0% to 4.6% (median: 0.3), respectively. CD41a expression level was found to be lower than CD61 expression level in all type 1 GT patients. Expression levels of CD41a, CD42b, CD61, and CD41a/CD61 in type 2 GT patients were 14.1% to 18.9% (median: 15.8), 93.5% to 97.3% (median: 94.6), 53.2% to 98.5% (median: 62.9), and 12.5% to 18.7% (median: 13.6), respectively. CD41a expression level was found to be lower than CD61 expression level in all type 2 GT patients as well. Expression levels of CD41a, CD42b, CD61, and CD41a/CD61 in type 3 GT patients were 48.6% to 98.2% (median: 87.2), 94.1% to 98.3% (median: 96.9), 57.1% to 85.1% (median: 64.8), and 35.4% to 84.8% (median: 61), respectively. In contrast to type 1 and type 2 GT patients, CD61 expression level was lower than CD41a expression level in this patient group. 

**Figure 2:**
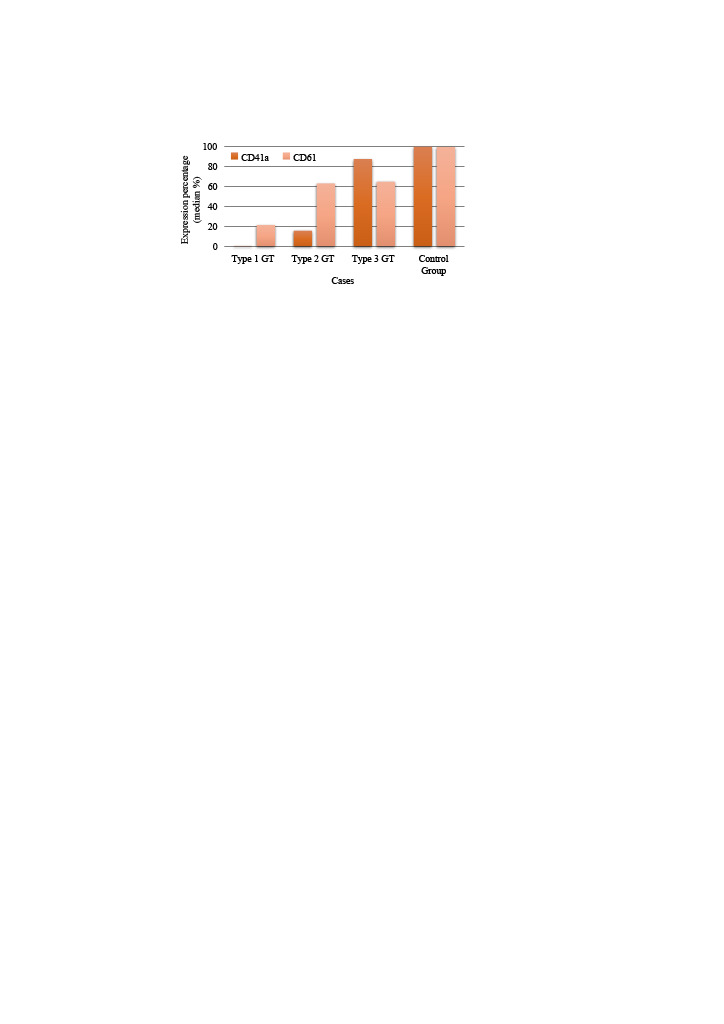
Distribution of CD41a and CD61 expression levels in GT subgroups and control group.

The distribution of CD41a and CD61 expression levels in patient and control groups were shown in (Figure 2).

## 4. Discussion

CD41a and CD61 receptors on the platelet surface are adhesion molecules involved in the platelet aggregation process. GT is a hereditary platelet disorder characterized by bleeding diathesis as a result of a defect in the synthesis of these receptors (failure to synthesize by mutation or closure with autoantibody) [17]. In our study, epistaxis (71.9%) and easy bruising (68.8%) were among the most common presenting symptoms. Menorrhagia was found to be one of the most common clinical findings in adolescent girls. These findings were similar to the studies in the literature [17–19].

In GT, the incidence of severe bleeding in general decreases with age [20]. No correlation between the levels of GP IIb/IIIa and severity of bleeding was reported [7,21]. In a study by Kutlubay et al. in 2012, out of 15 Turkish GT cases 7, 6 and 2 of the patients were compatible with type 1, type 2, and type 3, respectively [22]. In 2015, Tokgöz et al. reported that out of 20 Turkish GT patients, 18 and 2 cases were compatible with type 1, and type 2, respectively [23].

In the present study, flow cytometry analysis showed that 12 out of 23 families were consistent with type 1 GT (52.2%), 4 were consistent with type 2 GT (17.4%), and 7 were consistent with type 3 GT (30.4%). Since type 1 GT is seen in 52.2% of the GT patients, type 1 is considered to be the most common subgroup in Turkey, followed by type 3 and type 2 that is seen in 30.4% and 17.4%, respectively. The data confirmed to the previous studies from Turkey that type 1 GT was most common in Turkish patients [22,23]. Our results were in agreement with the result of Kannan et al. study and Hassan et al. study that revealed the highest frequency in type 1 GT (64% and 86%) followed by type 3 GT (24% and 11%) [24,25].

In our study CD41a expression level was found to be lower than CD61 expression level in all type 1 and type 2 GT patients. However, CD61 expression level was lower than CD41 expression level in type 3 GT patients. These findings were similar to the studies in the literature [26]. 

Studies have shown that GT is more common in women than men probably due to more females were diagnosed due to heavy menstrual bleeding. Approximately 58% of GT cases were women and 42% were men [12]. However, 18 of the young 32 GT patients were male (56.3%) and 14 were female (43.7%) in this study. Records of our patients indicate that diagnosis was made in the first and second decade of life. 

GT is more common among ethnic groups, such as Iraqi Jews, Palestinians, and French Gypsies, with high consanguinity rates, although they can be seen in all communities around the world. Disease was seen in about 150 out of 300 French Gypsies [15,16]. There was history of consanguinity in 86.9% of our families which was consistent with the frequency of 90% reported in a previous study from Turkey [23]. 

In addition to flow cytometry in the identification of GT; western blot, dot blot, and ELISA methods have also been reported [27–29]. Farsinejad et al. found that CD61 expression level was higher than CD41 expression level in the western blot analysis [27]. Vijapurkar et al. emphasized that the dot blot method could be used to diagnose type 1 GT but this method is not useful in defining other subgroups [28]. Lobo et al. developed the ELISA method for detecting GT. They could detect type 1 GT subgroup but failed to identify the other subgroups by ELISA [29].

Especially due to consanguineous marriages, GT with various glycoprotein levels may be detected. As a result of the flow cytometry analysis of the present study with the highest GT patient population in Turkey, type 1 GT patients were the most common subgroup. In the determination of the GT subgroups; especially in the detection of type 3 GT, flow cytometry is the most sensitive glycoprotein analysis method. In addition to light transmission aggregometry, CD41a/CD61 study by flow cytometer confirms diagnosis when mutation analysis cannot be performed.

## Informed consent

Ethical approval of the study was obtained from Ethics Committee of Erciyes University Faculty of Medicine (Approval number: 2013/271). Clinical and laboratory data and consent forms of the patients were also obtained from treating centers. The study was carried out in accordance with the principles of the Helsinki Convention on Human Rights.
